# Novel Palladium Complex: Cytotoxicity against Cisplatin-resistant K562 Cells

**DOI:** 10.22037/ijpr.2019.1100714

**Published:** 2019

**Authors:** Ali Jahanian-Najafabadi, Mina Mirian, Fatemeh Rohani, Kazem Karami, Mahboubeh Hosseini Kharat, Hojjat Sadeghi-Aliabadi

**Affiliations:** a *Department of Pharmaceutical Biotechnology, School of Pharmacy, Isfahan University of Medical Sciences, Isfahan, Iran. *; b *Department of Pharmaceutical Chemistry, School of Pharmacy, Isfahan University of Medical Sciences, Isfahan, Iran.*; c *Department of Chemistry, Isfahan University of Technology, Isfahan, Iran.*

**Keywords:** K562 cells, Cisplatin resistance, Palladacyclic complex, MTT assay, Apoptosis assay

## Abstract

Today, development of resistance to anticancer drugs (including cisplatin) is noticed as a major problem. Recently several studies demonstrated that palladium complexes showed remarkable cytotoxic effects against K562 cell line and could be used efficiently for treatment of many human cancers including leukemia. Hereof, K562 cells were made resistant to cisplatin using increasing concentration of cisplatin up to 4.5 mM and then cytotoxic effect of synthesized palladium complex was evaluated on this sub-line using MTT assay. Annexin V/PI staining using flow cytometry and scanning electron microscopy (SEM) were performed to find out the mechanism of the observed cytotoxicity. Results indicated that tested compounds had a noticeable cytotoxic effect on K562 cells 80 times more than cisplatin. Palladium complex also showed significant cytotoxicity on resistant K562 sub-line. Flow cytometry and SEM results revealed that these compounds exert their cytotoxic effect via apoptosis and it could be concluded that the novel synthesized palladium complex might be a good candidate for replacing cisplatin in case of treatment of cisplatin resistant tumors.

## Introduction

Cancer is one of the most important health problems in developed and developing countries in the word, while today in the United States one of four deaths is due to various types of cancers (1, 2).

Surgery, radiotherapy, and chemotherapy are 3 main treatments of different cancer types (3).

**Figure 1:**
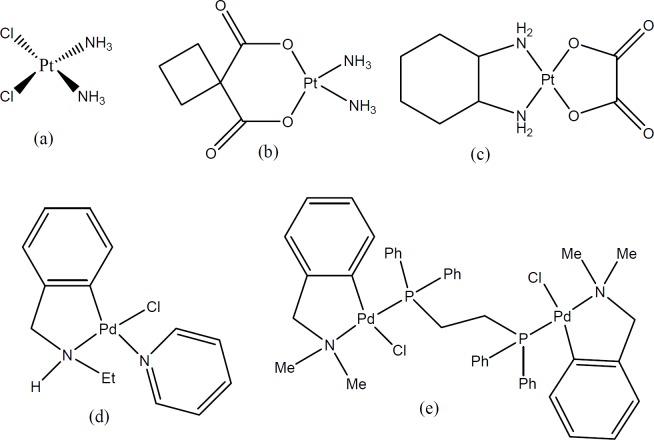
Structures of (a) cisplatin, (b) carboplatin, (c) oxaliplatin, (d) synthesized mono-nuclear and (e) the biphosphonic palladacycle complexes used in this study

Cisplatin is an effective anticancer chemotherapeutic agent used in the treatment of many human

cancers. However its clinical use is limited because of its severe side effects including

neurotoxicity nephrotoxicity, and nausea. Furthermore acquired or innate resistance to cisplatin and its analogues (carboplatin and oxaliplatin; Figure 1) reduced their usage in clinical treatments (4-6). The recurrence of cancer after cisplatin application or other platinum (Pt(II)) complexes in small cell lung cancer is almost 95 percent. In the case of head and neck cancers, which cisplatin is the first choice of treatment, only 20 to 30 percent of patients showed a good response to the treatment (7). Metal complexes including ruthenium, gallium, gold, and palladium are other nonplatinum complexes with anti-tumor activity that have been recently entered in clinical trials (8).

Palladium(II) complexes have been introduced as potential anticancer agent during last decades. Because of their structural and thermodynamic similarities to Pt(II) complexes they could be good alternatives for cisplatin in treatment of recurrent cancers (9, 10). Many studies indicated that palladium complexes have an attracted particular attention due to their cytotoxic activity and showed fewer side effects such as nephrotoxicity in comparison to cisplatin (11). According to our previous studies, mono nuclear biphosphonate palladacycle complexes (Figure 1d) showed a noticeable cytotoxic activity against Hela HT-29, and K562 cell lines. Bisphosphonate analogs displayed higher cytotoxic effect than the other congeners (12).

We previously reported synthesis and evaluation of cytotoxic activity of some new palladiumderived complexes which showed reasonable killing activity on cancerous cells (12). Thus in the present study, first we developed a cisplatin-resistant K562 cell line, and then evaluated the cytotoxic activity of the selected compound on these cells. Finally, the mechanism of the induced cell death was evaluated by flow cytometry.

## Experimental

In this study, cisplatin was purchased from Sigma-Aldrich Co (USA). MTT was obtained from Merck Chemical Company (Germany). K562 (leukemia cancer cell line) was purchased from Pasteur Institute of Iran, (Tehran, Iran). Palladacycle complex was synthesized by our colleagues in Isfahan University of Technology, Isfahan, Iran, as previously reported (12).

*Cell line maintenance*

K562 cells were cultured in RPMI 1640 medium (Bioidea, Iran) supplemented with 10% heatinactivated fetal bovine serum (Gibco, USA) penicillin (100 UµmL; Gibco, USA)streptomycin (100 μgmL; Gibco, USA) µL-glutamine (2 mM; Gibco, USA) in a highly humidified atmosphere with 5% CO2 at 37 °C. Cisplatin-resistant K562 cells were established in our laboratory by culturing K562 cells in the presence of increasing dose of cisplatin, starting from 0.01 μM and increased gradually up to a maximum concentration of 4.5 μM during 6 months (13). Afterwards, following 3-4 weeks maintenance in medium containing highest concentration of cisplatin the cells were seeded into a 96-well plate at a density of 100 cells well to grow and produce large colonies. Colonies were isolated and propagated as resistant subline. Experiments were performed on this subline 2-3 weeks after cisplatin removal and their cultivation in drug-free medium.

*Confirmation of K562 resistant cells*

One-hundred and eighty µL of prepared resistant K562 cells (1 × 10^5^ cells µmL) were seeded into 96-well plate and incubated for 24 h. Then, the cells were exposed to various concentrations of cisplatin including 0.5, 1, 2, 4.5, 6, 8, and 10 μM and incubated for 48 h. To evaluate cell survival, 20 µL of MTT solution (5 mg mL in PBS) was added to each well and incubated for 3 h. Then, the media were carefully replaced with 150 µL of DMSO to dissolve insoluble formazan crystals.

The formazan absorption was measured at 570 nm using an ELISA plate reader (Statfix 2000, Awareness, USA). The percentage of cell survival was calculated according to the following equation:

Cell survival %=mean abs of treated sample-mean abs of blank mean abs of negative control-mean abs of blank ×100

*Cytotoxic evaluation of palladium complex on resistant K562 cells*

Cytotoxic assay was performed as reported previously (14). Briefly, 180 µL of resistant K562 cells (1 × 10^5^ cells µmL) were seeded into 96-well plate and incubated for 24 h. Then, 20 µL of different concentrations of cisplatin (2.5, 5, 10, 15, 20, 30 and 40 µM) and palladium complex (0.05, 0.1, 0.2, 0.5, 1, 1.5, 2, 2.5 and 5 µM; dissolved in 10% DMSO) were added to each well and incubated for 48 h. The percent of cell survival was calculated as mentioned above.

**Table 1:** The IC50 values of cisplatin and synthesized palladium complex against K562 (S) sensitive and (R) resistant to cisplatin. Errors reported in SD

**Cell line**	**Compounds**	**IC** **50 ** **(µM)**
K562 (S)	Cisplatin	10 ± 2
	Palladium complex	0.0625 ± 0.01
K562 (R)	Cisplatin	>20
	Palladium complex	0.25 ± 0.05

**Figure 2:**
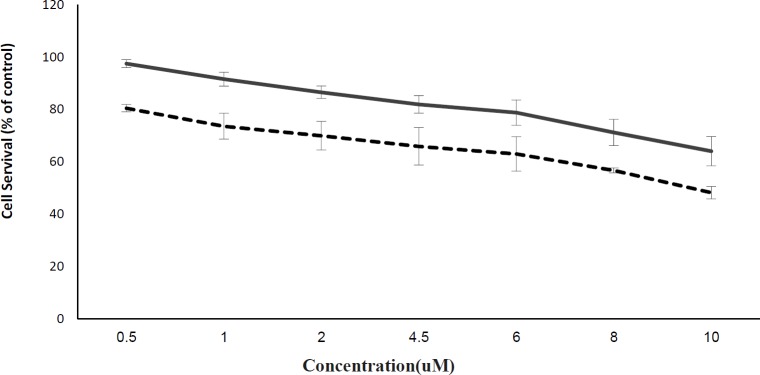
The effect of cisplatin against sensitive (dashed line) and resistant K562 (solid line) cells. K562 cells were exposed to increasing concentration of cisplatin (0.01 to 4.5   ) during a period of 6 months. Both sensitive and resistant K562 cells (5 × 10^5 ^cell mL) were exposed to different concentrations of cisplatin for 48 h and viability were evaluated using MTT assay. Data are represented as mean   SD (*P* < 0.05, n = 3)

**Figure 3:**
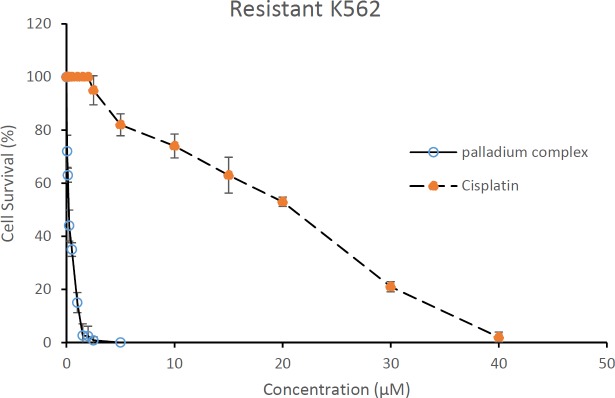
Cytotoxic effect of cisplatin (dashed line) and palladium complex (solid line) on the resistant K562 cells. The resistant K562 cells (5 × 10^5 ^cell mL) were exposed to different concentrations of compounds for 48 h and cells viability were evaluated using MTT assay. Data are presented as mean   SD (*P* < 0.05, n = 3)

**Figure 4:**
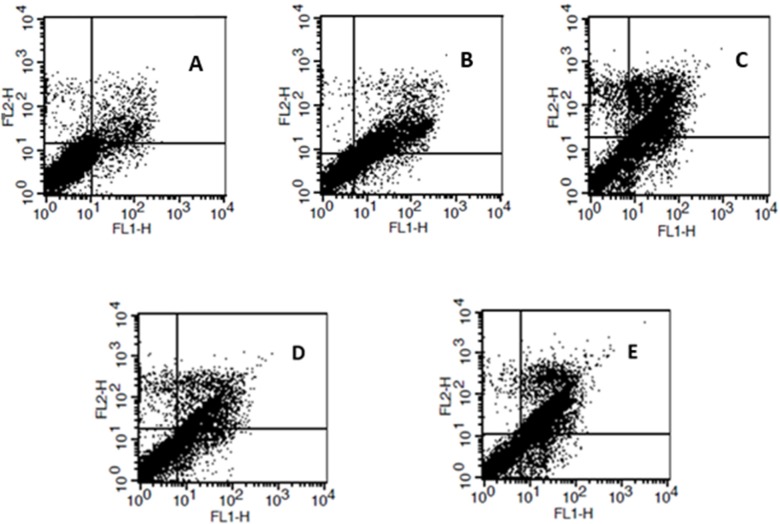
Analysis of cell death mechanism in K562 cells by flow cytometry. (A) Control non-treated cells, cells were treated with (B) 5   , and (C) 10    of cisplatin, or (D) 0.125    and (E) 0.25    of the palladium complex for 12 h, and then stained with Annexin V-FITC and PI

**Figure 5:**
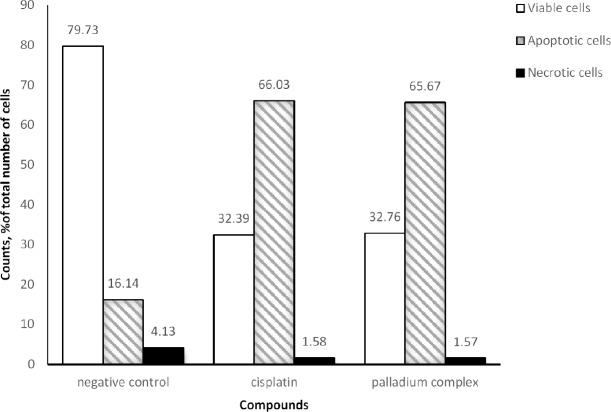
Results of Annexin V-FITC PI assay for evaluation of apoptosis and necrosis induction in K562 cells. Cells were treated by cisplatin (5   ) and the palladium complex (0.25   ) for 12 h

**Figure 6:**
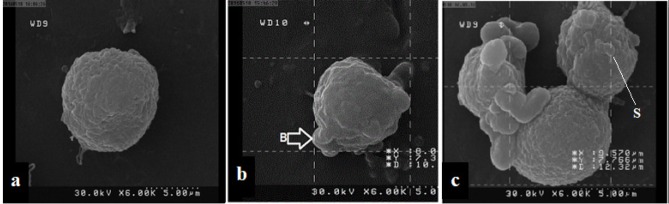
SEM micrographs of K562 surface cells treated with palladium complex. (a) Untreated K562 cells illustrated the restoration of specific morphological appearance of cancer cells. (b) and c treated K562 cells with palladium complex, 6 and 12 h after exposure respectively, showed obvious morphological changes in order to typical apoptosis, including cell membrane blebbing (B) and cytoplasmic extrusions (S)

*Apoptosis assay*

In order to evaluate cell death mechanism induced by the palladium complex, apoptosis assay was performed by flow cytometer according to the manufacturer protocol using Annexin VFITC/PI staining kit (Roche, Germany). In this regard, K562 cells were treated for 12 h with different concentrations of the palladium complex (0.25 and 0.125 µM), and cisplatin (10 and 5 µM) as positive control as mentioned already (15). Briefly, the cells were washed in PBS by gentle shaking or pipetting up and resuspended in 200 µL of binding buffer. According to kit instruction, 5 µL of Annexin V-FITC solution was added to 195 µL of the K562 cell suspension (5 × 10^5^ cellµmL), and incubated for 10 min at room temperature. Then, the cells were washed with 200 µL of binding buffer, and again resuspended in 190 µL of this buffer. Finally, 10 µL of Propidium Iodide solution (20 µgµmL) was added to the cells and subjected to flow cytometry assay using BD FACScalibur Partec™ instrument. BD CellQuest Pro software (version 5.1) was used for interpretation of folw cytometry results.

*Scanning electron microscopy (SEM)*

In this part, palladium complex was applied to membrane blebbing induction which occurs generally during apoptosis. K562 cells were incubated with palladium complex (0.25 µM) for 6 and 12 h. Then, the cells were trypsinized with 0.25% trypsin*/EDTA *(Gibco, USA) and centrifuged at 300 g for 5 min. 

The cells were fixed in 2.5% glutaraldehyde for 4 h at room temperature. Dehydration process was carried out in alcohol ascending grades (50, 70, 80, 90, and 100% V/V) for 10 min. Then, they brought to the critical point of drying by the critical point dryer (S4160, Hitachi, Japan) for 30 min. The cells were fixed on a metal SEM stub and sputter coated in gold using SEM coating unit (E5100 Polaron, UK). The coated specimen was evaluated via scanning electron microscopy (JOEL 64000, Japan) at an acceleration voltage of 15-25 KV.

*Statistical Analysis*

Cell toxicity results were expressed as the mean ± standard deviation (SD) and calculated by Microsoft SPSS 21 software. Data were analyzed using one-way ANOVA followed by LSD (Posthoc) to evaluate the differences between groups*. P *< 0.05 was considered as statistical significance for all data.

## Results

*Verification of resistant K562 cells*

To confirm cisplatin resistance of K562 cells, different concentrations of cisplatin were evaluated against sensitive and resistant cells for 48 h and cell viability was measured by MTT assay. As shown in Figure 2, the viability of sensitive K562 cells was less than 50% at concentrations µ10 µM, while it was more than 65% for the resistant cells.

*Cytotoxic evaluation of cisplatin and palladium complex on resistant cells*

The cytotoxic activity of cisplatin (Figure 3) was tested against resistant K562 cells. As shown in Figure 3, cisplatin IC_50_ was µ20 µM which is 2 time higher than its IC_50_ against sensitive K562 cells.

As seen in Figure 3, the tested palladium complex with an IC_50_ of 0.25 µM demonstrated noticeable cytotoxic effects against resistant K562 cells. According to these results the palladium complex was approximately 160 and 80 times more potent than cisplatin against the sensitive and established resistant K562 cells, respectively (Table 1).

*Induction of apoptosis in K562 cells with the palladium complex*

Pro-apoptotic effect of cisplatin and the palladium complex against K562 cells was tested by Annexin V-FITC PI staining as illustrated in Figures 4A-4E. The tested palladium complex like cisplatin, showed its cytotoxic mechanism via apoptosis induction. Non-treated cells were assumed as negative control whereas the cisplatin treated cells were considered as positive control.

As seen in Flow cytometry results (Figure 5) in negative control almost 80% of cells are viable and only 16% of them are apoptotic. However, in the case of treated cells percent of apoptotic cell was about 66% that confirmed apoptosis as cell death mechanism of both cisplatin and the palladium complex. It must be noted that the observed amount of the apoptotic cells was related to the different concentrations of the two tested compounds i.e 5 µM and 0.25 µM for cisplatin and the palladium complex, respectively.

*Palladium complex affected ultrastructur*e *of K562 cells*

The surface ultrastructure of K562 cells incubated with palladium complex was assessed using SEM. Untreated K562 cells have shown a restoration of the typical morphological appearance of chronic myelogenous leukemia cell line (Figure 6a). The effect of palladium complex on K562 cells, 6 and 12 h after exposure are shown in Figures 6b and 6c, respectively. Commentary of SEM electron-micrograph showing obvious morphological changes was similar to a typical cellular surface morphology of apoptosis, including cell membrane blebbing and separated apoptotic bodies.

## Discussion

Anti-cancer chemotherapeutic agents like cisplatin are widely used to treat different kinds of cancers (16). Today, the development of resistance to anticancer drugs (including cisplatin) is noticed as a major problem (17). Several mechanisms such as less susceptibility to natural killer (NK) cells in cisplatin resistant cells, decreasing cisplatin accumulation, increasing drug inactivation and deficiency in the apoptotic pathway were shown to be the most important reasons of drug resistance in cancer cells (18-20). Recently several studies demonstrated that palladium complexes had remarkable cytotoxic effects and could be used efficiently for the treatment of many human cancers including leukemia (21, 22). Zareian *et al. *in a comprehensive review showed palladium complexes with different leaving ligands had noticeable cytotoxic activity very close or superior to cisplatin (23). In consistent with these finding, we showed that the palladium complex [Pd2 (C,N-N,N-dimethylaminebenzylamine)2 (μ1,2(diphenylphosphinoe)ethane)(Cl)_2_] had significant cytotoxic activity toward the K562 cells that was comparable to cisplatin (12).

Considering the previous results, in the present study K562 cells were made resistant to cisplatin by their cultivation in the presence of increasing doses of cisplatin to a maximum concentration of 4.5 µM during 6 months. Our method was more efficient than others where in a similar study prepared a cisplatin resistant sub-line of K562 cells in which the maximum resistible concentration was 3.3 µM. Percent cell survival of resistant and sensitive K562 cells after 48 h of exposure to cisplatin at a concentration of 4.5 µM was almost 82% and 66%, respectively (24). According to this data the percent of cell survival in the resistant cells was 1.3 fold higher than the sensitive cells. However, both sublines responded to increasing doses of cisplatin in a dose dependent manner. In addition, the resistant cells showed an IC_50_ higher than the IC_50 _obtained for nonresistant cells (10 µM). This increased cell survival confirmed resistance of the K562 cells to cisplatin. Nikounezhad *et al. *generate an ovarian cancer cell line resistant to cisplatin which exhibited at least 5.1 fold resistance to the drug (25). It seems that different cell lines show diverse patterns of resistance to cisplatin which may relate to the amount of GSH level in the cells (26). In the present study the palladium complexes were chosen because of their structural similarity to Pt (II) complexes. In consistent with our recent results, it was shown that palladium complexes despite their lower toxicity toward normal cells, are more potent against cancer cells in comparison with cisplatin (12, 27). Data obtained from MTT analysis in the present study showed inhibitory effect of palladium in low concentration against resistant cells which is comparable to cisplatin. The IC_50_ of the palladium complex was 0.25 µM while this value for cisplatin was 20 µM showing that the palladium complex is about 80 times more potent than cisplatin. Our results are consistent with others where they showed higher cytotoxic effect of palladium complexes in comparison to cisplatin (28). This data demonstrated that palladium complex had significant cytotoxic effect against the developed resistant K562 subline, and could be introduced as an alternative for cisplatin, of course, following further studies and confirmations.

In this study, we evaluated cell death mechanism induced by the palladium complex, with cisplatin as positive control against K562 cell line. K562 cells were treated at 0.25 µM, and 0.125 µM concentrations of the palladium complex, and 5 and 10 µM of cisplatin. Our results support our hypotheses and are consistent with the results of Tanaka M *et al*. where they tested a glycoconjugated Pd (II) complex against cisplatin resistant gastric cancer cells and showed that this complex was a potentially useful antitumor agent that executed its biological effects by inducing apoptosis (29). According to the flow cytometry results in the negative control (without any treatment) the percent of viable cells was almost 80%. For the positive control (5 µM of cisplatin), this value declined to 32% whereas the percent of apoptotic cells was about 66%. When 10 µM cisplatin was used, the cell survival and apoptosis percent was about 29% and 64%, respectively. As shown in Figure 5, treatment with the palladium complex resulted in 66% apoptotic cells. These results were confirmed by SEM. As shown in Figure 6 morphological characteristic changes showed that these compounds could potentially lead to an apoptotic phenomenon. SEM showed accurate changes in the cell surface such as erosion, formation of membrane blebs, and apoptotic bodies in the treated K562 cells. When these results were compared to the negative control, it could be concluded that the anticancer activity of the palladium complex was through the induction of apoptosis.

In conclusion, the newly synthesized palladium complex was cytotoxic against regular and cisplatin resistant K562 cell line mostly via apoptosis induction at a concentration remarkably less than cisplatin. So it could be said that these compounds are active against cisplatin resistant cell lines. Since we obtained promising results in this study, further *in-vitro *and *in-vivo *preclinical studies on evaluation of specific and non-specific cytotoxicities of the palladium complex will be demanded.
